# A case of enamel renal syndrome from a novel genetic mutation, multidisciplinary management and long-term prognosis

**DOI:** 10.48101/ujms.v129.10228

**Published:** 2024-09-13

**Authors:** Maria Erkapers, Carina Frykholm, Hans Furuland, Susanna Segerström, Andreas Thor

**Affiliations:** a Department of Prosthetic Dentistry, Specialist Clinic Kaniken, Public Dental Health Service, Uppsala, Sweden; b Department of Immunology, Genetics and Pathology, Uppsala University and Uppsala University Hospital, Uppsala, Sweden; c Department of Nephrology, Uppsala University and Uppsala University Hospital, Uppsala, Sweden; d Institute of Surgical Sciences, Department of Plastic and Oral & Maxillofacial Surgery, Uppsala University, Uppsala, Sweden

**Keywords:** Amelogenesis imperfecta, nephrocalcinosis, genotype, FAM20A, dental treatment, case report

## Abstract

**Background:**

The heterogeneous features of enamel renal syndrome (ERS) make diagnosis and treatment challenging. The main symptoms are disturbed amelogenesis and nephrocalcinosis. Bi-allelic likely pathogenic (LP) or pathogenic (P) variants in *FAM20A* have been associated with the syndrome since 2012. Affected patients often receive extensive dental treatment because of deviant orofacial morphology. However, knowledge about long-term prognosis and treatment guidelines are still lacking. The complex nature of ERS might endanger both dental and general health. The purpose of this article is to highlight the risks of overlooking the symptoms of the syndrome, and to discuss management strategies, surveillance and prognosis.

**Case presentation:**

We report the management of a case with suspected ERS after initial dental treatment elsewhere with no adjustment for the syndrome. Dental treatment was revised and followed for 8 years. Complementary medical examinations were conducted, and ERS was genetically confirmed, revealing homozygosity for a LP c.755_757del, p.(Phe252del) variant in *FAM20A*. The nephrological investigation revealed medullary calcium deposits, normal renal function and hypophosphatemia. Urine analysis revealed hypocitraturia and hypocalciuria. Accordingly, the patient now medicates with potassium citrate to decrease the risk of progressive renal stone formation.

**Conclusion:**

We herein describe a patient with confirmed ERS with an 8-year follow-up. Diagnostic delay until adulthood led to complicated dental treatment. The results of nephrological investigations are presented. The importance of dental and medical multidisciplinary management in syndromic disorders affecting the formation of the enamel is also exemplified. The dental prognosis after rehabilitation is likely affected by anatomical variations and patient cooperation. The prognosis for renal function seems to be good. However, lifelong surveillance of renal function is recommended.

**Registration:**

The ethics committee in Uppsala, Sweden, determined that ethical approval was not necessary in this case (2019-04835). Informed consent was obtained from the participant in writing and is documented in the medical records.

## Introduction

Amelogenesis imperfecta (AI) is a clinically and genetically heterogeneous group of inherited disorders that affects the structure and clinical appearance of the dental enamel (1). It is divided up into four different categories, according to the most used Witkop’s classification: hypoplastic, hypocalcified, hypomaturation, and a fourth combined form: hypomaturation/hypoplastic with taurodontia (2, 3). The inheritance patterns for AI are autosomal dominant, autosomal recessive, or X-linked. To date, 17 genes have been associated with non-syndromic AI and seven genes with syndromic AI (4, 5). Amelogenesis imperfecta is usually an isolated clinical finding, though almost one third of all AI cases are associated with a syndromic form of AI. In total, more than 70 genes have been associated with enamel abnormalities according to recent studies (5). This is important to address since dental problems can sometimes precede other clinical symptoms in syndromes. Syndromic AI displays a variety of intraoral deviations and associated symptoms, depending on the type of syndrome.

Enamel renal syndrome (ERS) is associated with hypoplastic AI (6) and numerous additional orodental deviations such as abnormal dentin, microdontia, spaced teeth, supernumerary premolars, localised aggressive periodontitis, thin alveolar bone, dental pulp calcifications, extensive crown and root resorption, aberrant root morphology, gingival fibromatosis, prolonged retention of deciduous teeth, impacted posterior teeth with hyperplastic follicle and open bite (7–12). Enamel renal syndrome is also associated with nephrocalcinosis, a condition characterised by deposition of calcium in renal tissue, usually in the renal medulla, and increased risk of recurrent urinary infections and renal tubular acidosis.

The prevalence of ERS is unknown (7). The syndrome is autosomal recessively inherited, and is caused by homozygous or compound heterozygous likely pathogenic (LP) or pathogenic (P) variants in the *FAM20A* gene (7). However, the diagnosis is not always confirmed through genetic testing in available publications (8).

The purpose of the present case report is to provide a complete description of a genetically confirmed ERS patient, a complete nephrological investigation and a detailed presentation of orodental features. The report also aims to highlight the importance of a multidisciplinary approach, with the dental team forming a vital component of the extended team for correct diagnosis and treatment. With the long-term follow-up presented, this case report constitutes a prognostic reference because of the lack of clinical trials evaluating the efficacy of specific nephrological and dental treatment protocols. This case report has been reported in accordance with the SCARE criteria (13).

## Case report

**Figure 1:**
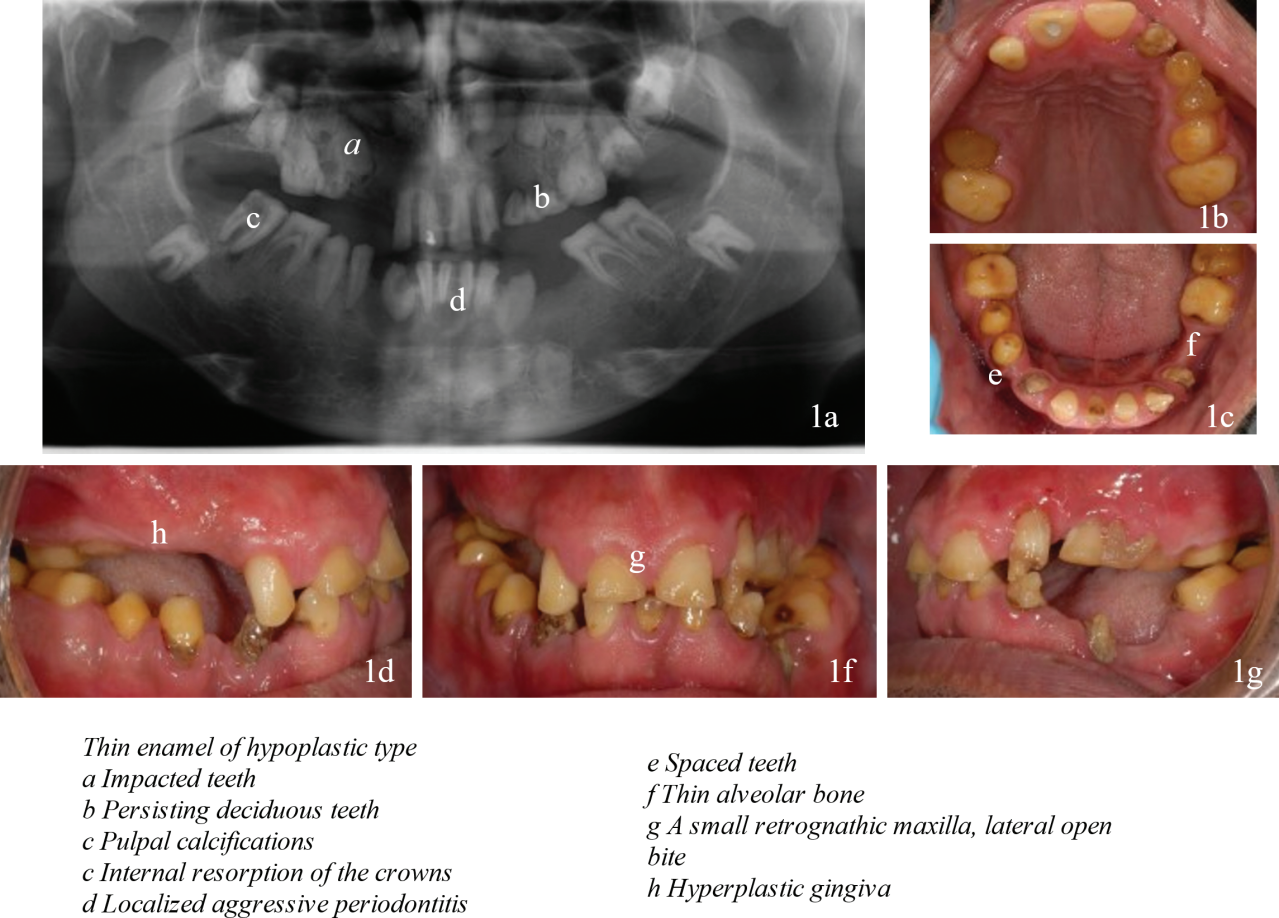
(a–g) Initial dental examination with orodental phenotypes associated with ERS.

A 27-year-old male was referred to Oral and Maxillofacial Surgery at Uppsala, Sweden, for dental care (Figure 1a–h).

### Family, social, and medical history

The patient grew up in Palestine and is of Arabic origin. His parents are first-degree cousins. Two of his nine siblings (one brother and one sister) have similar dental deviations. Five paternal half siblings are all unaffected. The patient reported no medical history except for a lifelong experience of oral dysfunction and aesthetic issues which had affected his quality of life. The patient reported smoking 10–20 cigarettes daily and no experience of alcohol.

### Dental diagnostic assessment and interpretation

The dentition included yellow and worn permanent and deciduous teeth surrounded by hyperplastic gingiva. The bite displayed a retrognathic maxilla, bilateral open bite, bilateral crossbite, elongated lower molars and thin alveolar bone where teeth were missing. The radiographical examination (orthopantomogram and intra oral conventional radiography) revealed thin enamel, impacted teeth, intra-pulpal calcifications, internal resorption, localised aggressive marginal periodontitis and apical periodontitis. A tentative diagnosis of syndromic AI was made.

After clinical investigation, the patient left and decided to have his dental treatment abroad.

Upon returning to Sweden, a new examination was carried out at the Department of Prosthodontics in Uppsala, Sweden.

### Revised dental diagnostic assessment and interpretation

**Figure 2:**
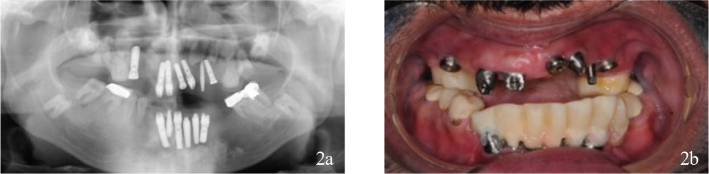
(a, b) Dental status after treatment abroad. Thirteen dental implants with a variety of designs and unfavourable placement had replaced teeth in both jaws. Interim prosthesis had been cemented on implant abutments and on teeth but was missing in the maxillae on examination. The molars in the mandibulae were restored with fixed dental prosthesis.

The patient reported malaise, oral pain and oral dysfunction after multiple extractions and extensive dental implant treatment abroad (Figure 2a, b). The brand of the implants could not be confirmed. The implants were distributed between the remaining teeth in both jaws, with different abutments and cover screws. Most implants lacked supporting bone, and some perforations impacted teeth causing implant mobility and inflamed mucosa.

The prosthodontic work carried out for the patient was non-functional at this time.

A tentative diagnosis of a hypoplastic form of AI associated with a syndrome was formed after clinical evaluation. Referrals were made to the departments of Oral and Maxillofacial Surgery, Clinical Genetics and Nephrology at Uppsala University Hospital for further investigations, diagnostics and multidisciplinary planning of dental treatment.

#### Dental treatment

**Figure 3:**
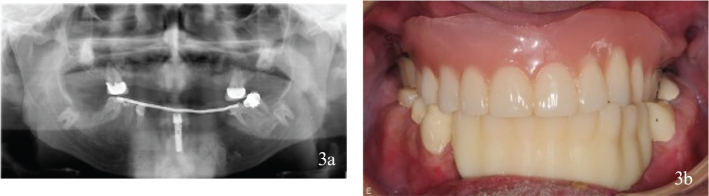
(a, b) Interim prosthetics. Interim prosthetics after removal of dental implants. A removable denture retained by fixed dental prosthesis on the molars in the maxillae and an interim acrylic prosthesis retained by teeth and the temporarily preserved implant in the mandible.

**Figure 4:**
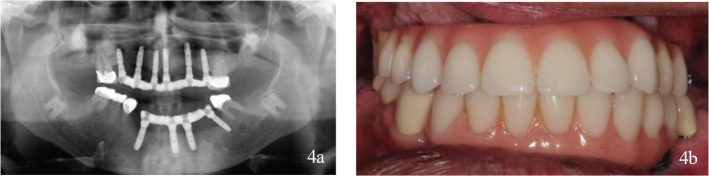
(a, b) Status after treatment. Six implants in the maxillae and four implants in the mandible (Astra Tech Implant System® Osseospeed® TX, Dentsply Sirona, Mölndal, Sweden) were provided with implant screw-retained fixed partial dentures with metal base and angulated screw channels (Heraeus Dental AB, Biomain®, Helsingborg, Sweden) on Uniabutments (Dentsply Sirona, Mölndal, Sweden). Teeth were restored with fixed dental prosthesis.

After initial periodontal evaluation and cessation of smoking, a surgical intervention with extractions of teeth and implants was performed. Bone replacement, including guided bone regeneration with bovine bone and collagenous membranes, was necessary because of large defects in the alveolar crest caused by implants that had penetrated retained teeth. One implant was temporarily preserved in the mandible for prosthetic retention purposes. The surgical procedure was followed by a healing period of 8 months, with interim prostheses (Figure 3a, b). Thereafter, new dental implants were installed in the maxillae and mandible. Finally, implant screw-retained fixed partial dentures were fitted in the maxilla and mandible, and teeth were restored with fixed dental prosthesis (Figure 4a, b).

#### Dental follow-up and outcome

**Figure 5:**
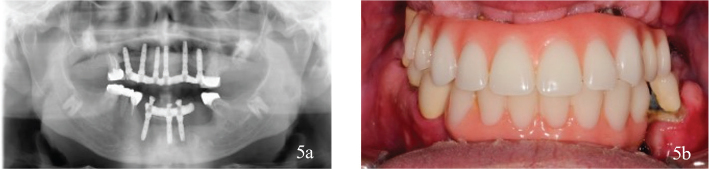
(a, b) Complications 8 years after treatment. Two teeth were endodontically treated. The distobuccal root on a molar in the maxillae had been resected. One implant in the mandible was lost because of loss of bone integration, and the fixed partial dentures was adjusted after the implant loss.

The patient was lost to follow-up for 3 years after dental treatment, but then returned to the specialist Prosthodontics Clinic for regular follow-ups. Eight years after dental treatment, two teeth had been endodontically treated, an upper molar had a distobuccal root removed, and one dental implant had been lost in the mandible. The implant-retained fixed partial denture (FPD) was modified accordingly (Figure 5a, b).

#### Genetic diagnostic assessment and interpretation

At the Department of Clinical Genetics, Uppsala University Hospital, a clinical assessment for dysmorphic features was carried out and a pedigree of the family was constructed. Because of parental consanguinity and several affected siblings, an autosomal recessively inherited condition was suspected. No dysmorphic features were seen in the patient apart from micrognathia and dental abnormalities. The study subject reported no photophobia. The skin was normal, without hypopigmentation. No nail or hair abnormalities were noted. Renal imaging, for syndromic assessment, showed nephrocalcinosis. A clinical diagnosis of ERS was established, followed by referral to the Nephrology Clinic at Uppsala University Hospital.

#### Genetic follow-up and outcome

The association of *FAM20A* gene to ERS was known from literature at the initial visit, but the gene was not available for clinical genetic testing (7). However, 6 years later, blood DNA was sent for genetic testing with a panel of genes described in amelogenesis and dentinogenesis imperfecta (16 genes, Blueprint Genetics, Helsinki, see supplement for full gene list). The *CLDN19* gene was added to the panel, for exclusion of familial hypomagnesemia with hypercalciuria and nephrocalcinosis, sometimes described with AI. Both sequencing and deletion/duplication analysis were included in the testing. No family members were available for genetic testing since they all lived abroad.

Genetic analysis revealed a homozygous LP c.755_757del, p.(Phe252del) variant in *FAM20A*. No other abnormalities were detected in the other 16 genes (including the following AI genes: *AMELX*, *CPR68*, *DLX3*, *ENAM*, *FAM83H*, *KLK4*, *LAMB3*, *MMP20*, and *SLC24A4*) or in *CLDN19*. The variant in *FAM20A* was considered LP since it had only previously been described in trans with another pathogenic variant in two siblings with ERS (14). A functional study that included this variant indicated that a deletion of phenylalanine in codon 252 had similar functional consequences on protein level as truncating mutations (11).

#### Renal diagnostic assessment and interpretation

Computed tomography (CT) of the kidneys and DXA bone density test (dual energy x-ray absorptiometry) were performed. The following blood samples were analysed: p(plasma)-calcium, p-phosphate, p-magnesium, p-bicarbonate, p-creatinine, eGFR (glomerular filtration rate), p-alkaline phosphatase, p-parathyroid hormone (PTH), p-25-OH-vitamin D, p-1,25-(OH)2-vitamin D, and p-FGF-23 (fibroblast growth factor 23). Urinary samples were u (urine)-calcium, u-phosphate, u-citrate, u-oxalate, u-urate, u-albumin/creatinine ratio, and u-magnesium.

#### Renal follow-up and outcome

**Figure 6:**
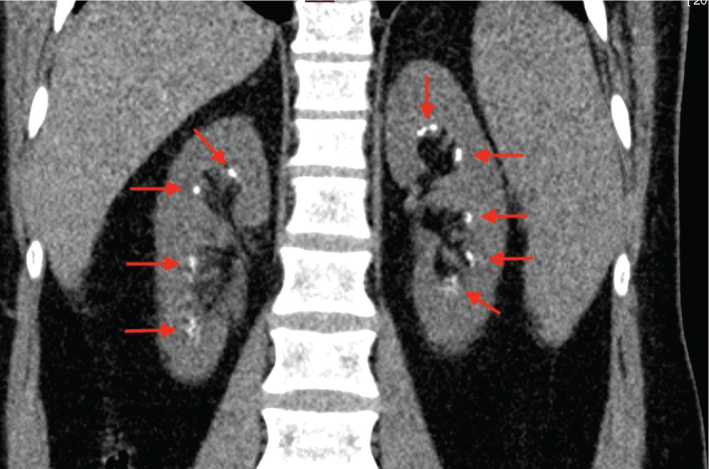
Bilateral nephrocalcinosis. Bilateral nephrocalcinosis in computed tomography (red arrows).

The first CT scan of the kidneys showed bilateral multiple small medullary calcium deposits in the renal calyces consistent with nephrocalcinosis (Figure 6). In a later CT scan after 6 years, the degree of nephrocalcinosis had not changed. A DXA bone densitometry showed normal values: T- and Z-scores were -0.6 and 0.3, respectively in the lumbar spine, and -0.4 and -0.1, respectively in the hip. The blood samples showed marked hypophosphatemia, borderline elevated p-alkaline phosphatase, slightly elevated p-PTH and p-FGF-23, low p-25-OH-vitamin D, hypocalciuria and hypocitraturia (Table 1). Plasma levels of calcium, magnesium, urate, bicarbonate, creatinine, eGFR, and 1,25-OH^2^-vitamin D and urinary levels of magnesium, urate, oxalate, and albumin were normal.

**Table 1:** Plasma and urine levels.

Plasma and urine samples	Patient value	Ref. value
p-phosphate	0.45 nmol/L	0.7–1.6
p-creatinine	101 μmol/L	60–105
eGFR	> 60 mL/min	> 60
p-alkaline phosphatase	1.9 μkat/L	0.6–1.8
p-PTH	11.4 pmol/L	1.6–6.9
p-25-OH-vitamin D	26 pmol/L	75–250
p-FGF-23	59 ng/L	10–50
u-calcium	< 0.5 mmol/d	2.8–8
u-phosphate	25.9 mmol/d	< 38
u-citrate	0.95 mmol/L	> 2

Presently, the patient has regular check-ups at the Nephrology Unit. The renal function has remained unaltered and normal. The blood and urine values have remained at the same levels. Because of low urinary citrate, potassium citrate was prescribed to increase urinary citrate levels, which decreases the risk of progressive renal stone formation. Seven years after the initial investigation, the patient suffered his first kidney stone attack. Increased water intake and intensified potassium citrate treatment were commenced.

## Discussion

This case report describes the complex nature of ERS. It also exemplifies the importance of multidisciplinary involvement for complementary investigations, diagnoses, treatments and follow-up examinations. In this case, ERS was genetically confirmed with a homozygous LP variant; Phe252del, in the *FAM20A* gene. To our knowledge, this genotype has not been described previously. Extensive analyses were done regarding the renal diagnostic assessment including the phosphate regulating hormone FGF-23 and generated a vital treatment for the patient. This report also illustrates compliance difficulties and how they impede dental procedures and nephrological long-term prognosis.

Enamel renal syndrome is associated with highly variable orofacial anatomy, and the successful management of this case cannot therefore be applied to all ERS individuals, but some important clinical experiences will be mentioned. An individualised dental treatment plan should be drawn up for every unique case, where dental and anatomical variations are considered through a multidisciplinary approach. It is desirable to preserve teeth if possible and apply fixed dental prosthesis when needed, to delay extractions. Lost teeth can be replaced with conventional fixed, removable or implant-retained prostheses. Implant treatment in AI patients requires careful planning because of anatomical variations, and guided bone regeneration is often necessary for an optimal result (15).

As in the presented case, patients with AI often experience aesthetic issues, dental sensitivity and oral dysfunctions which affect quality of life (16–18). Dental rehabilitation improves their quality of life (19), but the dental restorations need maintenance as severe conditions often require lifelong dental treatment. The prognosis of crown therapy on patients with AI is generally good (20). However, dental restorations on patients with hypomineralised and hypomatured AI have shorter longevity than those with hypoplastic AI (21). Prognosis also depends on the type of treatment, the degree of dental implication from AI and the skills of the practitioner.

In this case, the initial dental treatment carried out elsewhere was not well planned. It would have been desirable to postpone dental implant treatment in this patient, because of the presence of impacted teeth affecting the dental implant’s long-term prognosis.

The revised dental treatment was extensive, and less invasive treatment alternative was therefore considered and discussed with the patient. However, he opted for the more extensive treatment.

The unusually high proportion of endodontic treatment needed is in accordance with Dellow et al. (9). This finding suggests that ERS patients might have a higher risk of endodontic complications after fixed prosthodontic treatment.

Because of its rarity, ERS is under-recognised by many dentists and physicians. Clinical similarities between isolated AI and ERS often result in an AI diagnosis, but no further investigations are carried out to exclude syndromic AI as ERS (3, 17, 22). To date, 24 genes have been described in association with AI, including seven genes associated with syndromic AI according to Witcop’s classification. New genetic methods have revealed that more than 70 genes of importance for enamel formation are predicted to exist (5). In this case, a homozygous c.755_757del, p.(Phe252del) variant in the *FAM20A* gene has been described for the first time. The variant was previously described in two siblings together with a truncated mutation on the other allele (14). A checklist for associated manifestations/symptoms (ectodermal findings, short stature, visual problems) for the dentist would be feasible to identify patients who need referral to a clinical geneticist. This also highlights the need for dental assessment in many syndromes in which other symptoms are more prominent.

Early diagnosis of ERS and referral to a nephrologist is essential to prevent clinical events of renal stones and deterioration of renal function. Of 56 cases with *FAM20A*-related AI, the majority (45 cases) also had nephrocalcinosis (10, 12, 14, 23–28). However, only a few cases have led to terminal renal failure, thus the long-term renal prognosis seems to be good (9).

The metabolic alterations and risk factors for nephrocalcinosis and nephrolithiasis were hypocalciuria, a profound hypophosphatemia and hypocitraturia. Hypocalciuria has been reported in other ERS cases (9, 28, 29). It is surprising that the patient had hypocalciuria, since nephrocalcinosis is usually coupled with hypercalciuria. One hypothesis is that the *FAM20A* mutation affects the kinase involved in the phosphorylation of proteins causing biomineralisation, affecting calcium metabolism and transport (30). No kidney biopsy has been carried out in ERS, but *FAM20A* expression was detected in the renal tubules and Bowman’s capsule by immunohistochemistry in a mouse model (12).

Hypophosphatemia is only reported once previously (9). Urinary phosphate levels have been reported as both low and normal (29, 31). This is the first case in which phosphate regulating hormone FGF-23 has been measured in ERS. It is unclear whether the slightly increased level could also be part of the explanation for the hypophosphatemia, since hyperphosphaturia would then be expected (32).

Hypocitraturia is a well-known risk factor promoting nephrolithiasis, and has also been reported in a few other cases (28). Because of a recent acute attack of a kidney stone, more intensive therapy with potassium citrate was commenced. The study subject reported no side effects from his medication.

## Conclusion

This case illustrates the importance of referral of patients with hypoplastic AI with additional findings for genetic testing. A new genotype likely pathogenic homozygous c.755_757del, p. (Phe252del) variant, not previously published, in the *FAM20A* gene was detected. Hence, the diagnosis of enamel-renal-syndrome was confirmed. This led to a multidisciplinary management by specialists in Prosthodontics, Oral and Maxillofacial surgery, clinical genetics and nephrology.

This case also illustrates the importance of information on the treatment plan which demands dedication from the patient. Before starting treatment, risks in connection with surgical and prosthodontic treatment should be clarified and a lifelong maintenance should be expected because of the uncertain prosthetic prognosis.

Enamel renal syndrome patients should also have nephrological evaluation and lifelong check-ups, in which the renal prognosis for the nephrocalcinosis seems to be good.
